# Frequency evaluation of different extraction protocols in orthodontic treatment during 35 years

**DOI:** 10.1186/s40510-014-0051-z

**Published:** 2014-08-12

**Authors:** Guilherme Janson, Fábio Rogério Torres Maria, Roberto Bombonatti

**Affiliations:** Department of Orthodontics, Bauru Dental School, University of São Paulo, Alameda Octávio Pinheiro Brisolla 9-75, Bauru, SP 17012-901 Brazil

**Keywords:** Orthodontic treatment, Frequency of treatment protocols, Extraction vs non-extraction

## Abstract

**Background:**

Studies that show frequencies of different orthodontic treatment protocols can be used as valuable parameters in the interpretation of treatment tendency with time. The purpose of this retrospective study was to evaluate all orthodontic treatment planning conducted at the Orthodontic Department at Bauru Dental School, University of São Paulo, Brazil, since 1973, in order to investigate extraction and non-extraction protocol frequencies selected at each considered period.

**Methods:**

The sample comprised 3,413 records of treated patients and was evaluated according to the protocol choice, divided into 10 groups: Protocol 0 (non-extraction); Protocol 1 (four first premolar extractions); Protocol 2 (two first maxillary and two second mandibular premolars); Protocol 3 (two maxillary premolar extractions); Protocol 4 (four second premolars); Protocol 5 (asymmetric premolar extractions); Protocol 6 (incisor or canine extractions); Protocol 7 (first or second molar extractions); Protocol 8 (atypical extractions) and Protocol 9 (agenesis and previously missing permanent teeth). These protocols were evaluated in seven 5-year intervals: Interval 1 (1973 to 1977); Interval 2 (1978 to 1982); Interval 3 (1983 to 1987); Interval 4 (1988 to 1992); Interval 5 (1993 to 1997); Interval 6 (1998 to 2002); Interval 7 (2003 to 2007). The frequency of each protocol was compared between the seven intervals, using the proportion test (*P* < 0.05).

**Results:**

The results showed that 10 protocol frequencies were significantly different among the 7 time intervals.

**Conclusions:**

The non-extraction protocol frequency increased gradually with consequent reduction of extraction treatments. The four premolar extraction protocol frequency decreased gradually while the two maxillary premolar extraction protocol has maintained the same frequency of indications throughout time.

## Background

The decision to extract teeth or not and the number of teeth to be extracted can influence the final result of orthodontic treatment, including esthetics, occlusion, satisfaction of patients and their families, as well as the treatment time [1,2]. For many years the extraction decision has instigated much discussion and controversies, often linked to personal preferences than scientific criteria [3]. In the last decades, Orthodontics has experienced conceptual and technological changes influenced by dominant trends in each time. Extraction orthodontic treatment, as an actual and accessible alternative therapy also seems to be susceptible to moments of transition.

Retrospective studies [3–8] of extraction frequencies in orthodontic treatments are quite scarce and usually reflect the reality of North America or Europe. In this way, it would be interesting to verify if the extraction frequencies in other parts of the world are different from these places. Therefore, the purpose of this retrospective study was to evaluate the frequency of the different treatment protocols at the Orthodontic Department of Bauru Dental School, University of São Paulo, Brazil, during the last 35 years.

## Methods

The data was retrospectively obtained from 3,745 consecutively treated patients from the files of the Orthodontic Department at Bauru Dental School, University of São Paulo, from 1973 to 2007. Patient files (records, extraoral, and intraoral photographs, radiographs and study models) were sequentially evaluated and 332 (8.86%) were excluded, resulting in 3,413 cases in the sample. Exclusion criteria were patient transfer or treatment drop out, preventive and orthopedic treatment without following orthodontic treatment with fixed appliances and cases without complete records.

The sample consisted of 1,475 males (43.21%) and 1,938 females (56.79%) treated with an initial mean age of 13.76 years (SD ± 3.65; range 5.20 to 49.00 years), divided according to the treatment protocol: Protocol 0 (non-extraction); Protocol 1 (four first premolar extractions); Protocol 2 (two maxillary first and two mandibular second premolar or a variation to three first premolar and one mandibular second premolar extractions); Protocol 3 (two maxillary premolar extractions); Protocol 4 (four second premolar or a variation to three second premolar and one mandibular first premolar extractions); Protocol 5 (asymmetric extractions - three premolars or only one premolar); Protocol 6 (incisor or canine extractions); Protocol 7 (first or second molar extractions); Protocol 8 (atypical extractions) and Protocol 9 (patients with agenesis or previously missing permanent teeth). The frequency of these protocols were evaluated in seven 5-year intervals similar to Profitt [8]: Interval 1 (1973 to 1977); Interval 2 (1978 to 1982); Interval 3 (1983 to 1987); Interval 4 (1988 to 1992); Interval 5 (1993 to 1997); Interval 6 (1998 to 2002); and Interval 7 (2003 to 2007). The frequency of each protocol was compared among the seven intervals in order to identify some predominant trends at each tested period. One-phase and two-phase treatments and re-planned cases that included extractions in the new planning were also quantified. After 3 weeks, 30 patients were randomly selected, and their treatment protocols were re-evaluated by the same examiner to verify the intraexaminer error.

The study protocol was approved by the Ethics Committee on Human Research of Bauru Dental School, University of São Paulo.

### Statistical analysis

The frequencies of each protocol were compared among the intervals with the proportion test [9]. Results were considered significant at *P* < 0.05. These analyses were performed with Statistica software (Statistica for Windows version 7.0, Statsoft, Tulsa, Okla.).

## Results

**Figure 1:**
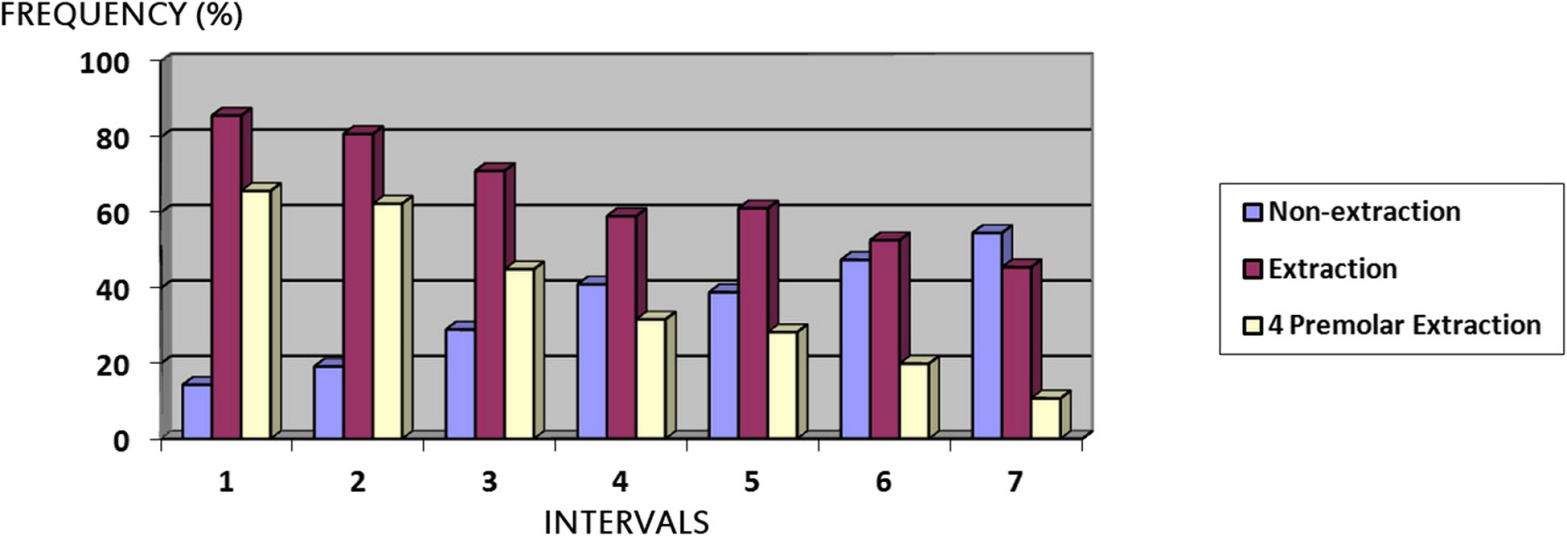
**Frequency of extraction and non-extraction treatment and premolar extraction protocol in all evaluated intervals.**

All 30 re-evaluated patient records presented complete agreement with the first observation, confirming the high reproducibility of the methodology. Figure 1 shows the frequency increase of non-extraction treatments and the reduction in the number of extraction treatments and four premolar extraction protocol in all evaluated intervals. Some protocol frequencies were significantly different among the seven intervals (Table 1). The two-phase treatments and the occurrence of re-planned case frequencies comparison are presented in Table 2.

**Table 1:** **Frequency of 10 treatment protocols in all evaluated intervals (proportion test)**

**Interval**	**Protocol**																				**Total**	
	**0**		**1**		**2**		**3**		**4**		**5**		**6**		**7**		**8**		**9**		***N***	**%**
	**Non-extraction**		**Four first premolar extractions**		**Two maxillary first and two mandibular second premolar extractions**		**Two maxillary premolar extractions**		**Four second premolar extractions**		**Asymmetric extractions - three premolars or only one premolar**		**Incisor or canine extractions**		**First or second molar extractions**		**Atypical extractions**		**Agenesis or previously missing permanent teeth**			
	***N***	**%**	***N***	**%**	***N***	**%**	***N***	**%**	***N***	**%**	***N***	**%**	***N***	**%**	***N***	**%**	***N***	**%**	***N***	**%**		
**1**	15	A	57	A	11	ABC	6	BC	1	BC	1	ABC	0	0	0	0	2	1.90	12	11.43	105	3.08
**(1973 to 1977)**		14.29		54.29		10.48		5.71		0.95		0.95										
**2**	28	AB	84	A	5	CD	5	AB	2	BC	1	AB	0	0	0	0	2	1.37	19	13.01	146	4.28
**(1978 to 1982)**		19.18		57.53		3.42		3.42		1.37		0.68										
**3**	91	B	90	B	40	A	34	BC	11	CD	4	A	1	0.32	3	0.96	14	4.46	26	8.28	314	9.20
**(1983 to 1987)**		28.98		28.66		12.74		10.83		3.50		1.27										
**4**	273	CD	170	B	34	BC	62	BC	7	BC	13	A	2	0.30	7	1.05	23	3.45	75	11.26	666	19.51
**(1988 to 1992)**		40.99		25.53		5.11		9.31		1.05		1.95										
**5**	491	C	281	B	63	BC	137	CD	13	BC	61	BC	6	0.48	10	0.79	61	4.83	139	11.01	1262	36.98
**(1993 to 1997)**		38.91		22.27		4.99		10.8		1.03		4.83										
**6**	290	DE	95	C	25	BCD	67	CD	2	AB	44	C	1	0.16	6	0.98	19	3.10	63	10.29	612	17.93
**(1998 to 2002)**		47.39		15.52		4.08		10.95		0.33		7.19										
**7**	168	E	23	D	4	D	23	BC	6	BC	21	C	0	0	2	0.65	10	3.25	51	16.56	308	9.02
**(2003 to 2007)**		54.55		7.47		1.30		7.47		1.95		6.82										
**Total**	1,356	39.73	800	23.43	182	5.3	334	9.78	42	1.23	145	4.24	10	0.29	28	0.82	131	3.83	385	11.28	3,413	100
**χ**	113.305		222.758		52.847		13.668		19.443		41.901		3.440		2.897		8.640		12.522			
***P***	0.0000*		0.0000*		0.0000*		0.0335*		0.0034*		0.0000*		0.7518		0.8215		0.1948		0.0512			

Different letters represent statistically significant differences in same protocol. *Statistically significant at *P* < 0.05.

**Table 2:** **Frequency of one-phase and two-phase treatments and re-planned cases in all evaluated intervals (proportion test)**

**Interval**	***N***	**One-phase**		**Two-phase**		**Not replanned**		**Re-planned**	
		***N***	**%**	***N***	**%**	***N***	**%**	***N***	**%**
**1**	105	104	99.05	1	0.95^A^	104	99.05	1	0.95^BC^
**(1973 to 1977)**									
**2**	146	145	99.32	1	0.68^A^	143	97.94	3	2.06^BC^
**(1978 to 1982)**									
**3**	314	309	98.73	5	1.27^A^	305	97.13	9	2.87^BC^
**(1983 to 1987)**									
**4**	666	636	98.05	30	1.95^AB^	653	98.05	13	1.95^BC^
**(1988 to 1992)**									
**5**	1262	1172	95.17	90	4.83^BC^	1226	97.15	36	2.85^BC^
**(1993 to 1997)**									
**6**	612	521	92.81	91	7.19^D^	587	95.91	25	4.09^AB^
**(1998 to 2002)**									
**7**	308	296	93.18	12	6.82^AB^	306	99.35	2	0.65^CD^
**(2003 to 2007)**									
**Total**	3,413	3,183	93.26	230	6.74	3324	97.39	89	2.61
		**χ**		101.272				12.726	
		***P***		0.0000*				0.0475*	

Different letters in two-phase treatment or in re-planned cases represent statistically significant differences. *Statistically significant at *P* < 0.05.

## Discussion

Because the sample consisted of all treated patients in the Orthodontic Department at Bauru Dental School, from 1973 to 2007, it would not be necessary to apply inferential statistical tests. Even though, to provide more mathematical precision, the Proportion Test was used to evaluate whether there was any significant difference in the treatment protocol frequencies between each time interval. This type of frequency distribution test is strongly influenced by the number of events observed. This explains why, in Table 1, the frequency of protocol 5 in interval 7 (6.82%) is statistically different from interval 3 (1.27%), but not different from interval 1 (0.95%).

In this study, the treatment protocols with (Protocols 1 to 9) and without extractions (Protocol 0) showed great statistically significant variation among the considered intervals (Figure 1 and Table 1). In the first interval, 1973 to 1977, 85.71% of cases were treated with some type of extraction protocol, demonstrating the influence of extraction dogmas at that time [10,11]. This tendency decreased, similar to other studies [6–8], until it reaches a frequency of 45.45% of cases with extractions in the last interval 2003 to 2007. These findings clearly demonstrate the great influence of extraction concepts on the percentage of cases treated with extractions in the 1960s and 1970s [10–13]. Since then, there has been a decrease of extraction treatment consequent to studies which showed relapses even in these cases [14,15], the possibility of protruding the mandibular incisors in some situations [16,17], the belief that there could be a relationship between extractions and temporomandibular disorders [18,19], and the possibility of treatments with interproximal stripping [20,21]. Technical changes also may have also influenced this decline, such as an increase in orthopedic appliances usage [22], maxillary expanders [23], as well as treatment in two phases [24,25].

The choice for four first premolars was for a long time the classic extraction protocol [10,26]. However, more recently, there are reports about treatment difficulty [27], greater treatment time [28], and risks of root resorption and periodontal problems [29], especially in adult patients. Investigations indicate that dental extractions tend to prolong treatment time, in general [30,31].

In this study, the frequency of treatments with four first premolar extractions (Protocol 1) decreased significantly, corroborating the findings of other studies [3,6–8] (Table 1). The frequency of two maxillary first premolar and two mandibular second premolar extraction protocol (Protocol 2) also demonstrated statistically significant differences among the evaluated intervals (Table 1). This reduction, also observed by others researchers [6–8], appears to have been influenced by the same historical reasons discussed for the four first premolar extractions protocol.

The two maxillary premolar extraction protocol (Protocol 3) showed a relatively stable frequency around 10% in most of the evaluated periods (Table 1). This fact can be interpreted as an increase in preference of this specific protocol due to drastic reduction of extraction treatment between 1973 and 2007 (Figure 1). In other investigations, frequencies ranged from 5% [5] to 22% [6]. Maxillary premolar extractions seem to be very useful in Class II malocclusion orthodontic treatment [18,28,32,33]. This treatment approach has a greater occlusal treatment success rate compared to four premolar extractions [32] and presents a shorter treatment time of complete Class II malocclusions [28]. This may minimize root resorption and iatrogenic effects, in addition to providing greater personal and financial benefits to patients [27,30].

The therapeutic choice of four second premolar extractions (Protocol 4) demonstrated a much-reduced frequency in all evaluated intervals (Table 1). Although it presents a small frequency, this protocol is usually used when anchorage can be lost, producing smaller impact on the soft tissues or in cases with moderate crowding [34,35]. Four premolar extractions frequency (Protocols 1, 2, and 4 together) decreased gradually from 65.72% (1973 to 1977) to 10.72% (2003 to 2007, Figure 1, Table 1).

The asymmetric extraction protocol of three premolars (Protocol 5 - two maxillary and one mandibular premolar) is indicated in Type 1 Class II subdivision malocclusion treatment [36]. Asymmetric extraction treatment provides an easier mechanics and better occlusal treatment success rate when compared to four premolar extractions [37] and less mandibular incisor and soft tissue retraction [38]. A variation of asymmetric extraction therapy in Class II malocclusions may include only one premolar extraction [39]. In this study, the initial frequency of indications of three premolar extractions was extremely low and increased to 7.19% between 1998 and 2002, when it was demonstrated to provide a better occlusal success rate than four premolar extractions in Class II subdivision malocclusions [37] (Table 1). While this frequency increased and remained stable over the last two intervals, there was a drastic reduction of the frequency of indications of extractions as a whole. Thus, similar to the protocol with two maxillary premolar extractions, there was an increase in the use of this protocol.

Protocols 6, 7, and 8 exhibited low frequencies without significant differences among them (Table 1). These findings seem to demonstrate certain stability of their indications in the evaluated periods. Consequently, it is speculated that these approaches are not susceptible to influences of prevalent philosophies. Mandibular incisor extraction frequencies were observed in the literature to be around 1% [5], 2.1% [3], 2.2% [7], and 2.5% [6] and molar extractions, 3.0% [3]. Mandibular incisor extraction should be considered in cases with tooth size discrepancy [40,41], although it may increase overbite and overjet [41,42]. It is also indicated when smaller posterior teeth mesialization is needed, to shorten treatment time, to produce smaller impact on the facial profile, for Class III malocclusion treatment and in cases with some periodontal problems [42–44]. Maxillary second molar extractions can be a valuable therapeutic approach which could lead to more stable results [45,46], facilitate first maxillary molar distalization, produce easier overbite correction [41,47] and smaller impact on facial profile, and present a smaller percentage of extraction spaces re-openings [48].

Protocol 9 included all cases with previous dental absences. It was considered that these patients should not be excluded from the study by the fact that similar circumstances happen in daily clinical routine, and should be statistically described. They were placed in a separate group because their previous condition could have influenced the treatment planning. The total mean frequency of these cases was 11.28%, and their frequencies in the several periods showed no statistically significant differences (Table 1).

Some authors suggest that a two-phase protocol in the treatment of Class II malocclusion provides best therapeutic results and greater stability [49,50]. However, this claim is very controversial because the influence of the orthopedic phase in the final clinical results is practically non-existent [51–55]. In this study, treatment was considered to have been conducted in two phases when patients had used only functional orthopedic appliances for more than 6 months for Class II malocclusion correction [24,49,56,57]. The frequency of two-phase treatment was of 6.74% and differed from other works that found a mean of 12% [7] and 20% [6]. Interval 6 (1998 to 2002) presented the highest frequency of two-phase treatment that was statistically different from the other periods. This greater frequency was probably due to the possible benefits that orthopedic correction could provide in Class II treatment, as was thought in the 1980s and 1990s (Table 2). On the other hand, also in Interval 6, there was a higher incidence of re-planned cases including extractions, probably due to failures or lack of patient compliance in the initial non-extraction approach [30,58] (Table 2).

### Clinical implications

Studies that show frequencies of different orthodontic treatment protocols can be used as valuable parameters in the interpretation of treatment tendency with time. In this way, the orthodontist can judge these tendencies and understand the actual reasons why accepted decisions have changed over years of orthodontic practice. Finally, these findings suggest the idea that modern orthodontist should not hold on paradigms without questioning them. It is up to him to be always updated and not to rely in dogmatic treatment approaches.

## Conclusions

The following conclusions are drawn from the study:

1. The non-extraction protocol frequency increased gradually from 14.29% (1973 to 1977) to 54.55% (2003 to 2007), with consequent reduction of extraction treatments from 85.71% (1973 to 1977) to 45.45% (2003 to 2007).
2. The four premolar extraction protocol frequency decreased gradually from 65.72% (1973 to 1977) to 10.72% (2003 to 2007), while the two maxillary premolar extraction protocol has shown the same frequency of indications in the same time period.
